# Anthropometric characteristics of four Polish children with mucopolysaccharidosis

**DOI:** 10.1186/1756-0500-6-246

**Published:** 2013-07-01

**Authors:** Lidia Perenc

**Affiliations:** 1 Institute of Physiotherapy, University of Rzeszów, Warszawska 26A, Rzeszów 35-205, Poland

**Keywords:** Mucopolysaccharidosis, Anthropometric Measures, Physical Growth, Case Report

## Abstract

**Background:**

Mucopolysaccharidosis relates to a group of seven prevalent types of disorders that are categorized on the basis of specific enzyme deficiency or the major symptoms, or both. A typical clinical presentation includes such symptoms and characteristics as short stature, facial dysmorphism, skeletal deformities, pulmonary dysfunction, joint stiffness and contractures, myocardial hypertrophy, neurological symptoms, and mental retardation.

**Case presentation:**

The purpose of this study was to perform a detailed anthropometric assessment in four cases of children with mucopolysaccharidosis (MPS) I and II types aged from 4 to 13 years from the Podkarpacie Province (south-eastern Poland). Anthropometric assessment included several parameters and indices related to body structure. All examined patients are characterized by severely disordered physical growth in comparison with the Polish norms presented in the reference charts.

**Conclusions:**

Examined children with MPS are characterized by especially low values relating to longitudinal and transversal parameters of body build. Anthropometric data could be used in early diagnosis of MPS and assessment of results of its treatment.

## Background

The term mucopolysaccharidosis (MPS) relates to a group of autosomal recessive metabolic disorders caused by the absence or dysfunction of the lysosomal enzymes involved in the degradation of glycosaminoglycals (GAGs). The MPS group comprises the seven prevalent types of disorder that are categorized on the basis of specific enzyme deficiency or the major clinical symptoms, or both [1]. A typical clinical presentation includes such symptoms and characteristics as short stature, facial dysmorphism, skeletal deformities, pulmonary dysfunction, joint stiffness and contractures, myocardial hypertrophy, neurological symptoms, and mental retardation. In most cases patients are symptomatic at the age of 2–4 years. Manifestation of the disease may be confused with other diseases such as inflammatory arthitides [2]. It should be mentioned that delays in diagnosis of MPS happen quite frequently and some children will suffer for some time with an unrecognized disorder. This kind of diseases are chronic and progressive even when they are treated or not. Some types of MPS are now treatable by means of enzyme replacement therapy and hematopoietic stem cell transplantation [3]. An important element of an exact assessment of the effects of treatment is the evaluation of the physical growth pattern in an individual patient. This kind of evaluation is based on anthropometric data.

The main study objective was to present and analyze selected anthropometric parameters and bodily proportions in four children with MPS I and II in order to highlight the distinctive manifestation of clinical symptoms of MPS in the dimension of physical development of the affected children. Results of the study may be used in the evaluation of the physical growth pattern of patients with MPS, its deviation in comparison with the reference charts and assessment of the effects of treatment.

## Case presentation

The study population consisted of 4 cases of children (3 males with MPS II) and 1 female (with MPS I) aged from 4 to 13 years. They represented the population of children with MPS from the Podkarpacie Province (south-eastern Poland) diagnosed at the time of examination (i.e. in 2010). The examination took place at the Department of Pediatrics of the Provincial Hospital No. 2 in Rzeszow. The author was invited to perform anthropometric studies of the 4 children diagnosed with MPS who participated in a special pharmacological treatment project. Three boys with MPS II were treated with Elaprase, and in the case of one girl with MPS I (Scheie syndrome) Aldurazyme was applied. All the children came from a rural social environment (Table 1). The anthropometric measurements were taking during an initial stage of the treatment programme. The following tools were used: anthropometric tape, physician scale, Martin anthropometer, and set of callipers (including skinfold calliper). All of them were used according to the standard anthropometric technique.

**Table 1:** Demographic information about children

**Case no.**	**1**	**2**	**3**	**4**
Age	11;1	13;7	12;1	4;4
Gender	male	male	male	female
Type of MPS	II	II	II	I
Social milieu	rural	rural	rural	rural

The examinations were based on the method of measurement and analysis of anthropometric parameters defined by Martin and Sailer [4]. Methodological principles recommended by the International Society for Advancement of Kinanthropometry were also followed [5]. The following parameters were used to evaluate longitudinal dimensionality: body mass, body height [B-v], trunk length [sst-sy], head and neck height [v-sst], lower extremity length [B-sy], and upper extremity length [a-adIII]. The following parameters were used to evaluate transversal dimensionality: shoulder breadth [a-a] and hip breadth [ic-ic]. The cephalometric parameters used in the study were: head length [g-op], head breadth [eu-eu], face breadth [zy-zy], face height [n-gn], nose height [n-sn] and nose breadth [al-al]. The circular dimensionalities were: chest circumference, head circumference and arm circumference. The chest circumference was measured through xiphoidale (xi) point. Skinfold thickness was measured with a constant-pressure skinfold caliper. The following parameters of skinfold thickness were measured: triceps skinfold, subscapular skinfold and suprailiac skinfold.

On the basis of anthropometric data the following indexes of proportion were calculated: trunk length index, head and neck for height index, shoulder-height index, pelvic-height index, upper extremity length index, arm length index, inter-extremity index, lower extremity length index, shoulder breadth index, pelvic breadth index, hip-to-shoulder index, global adiposity (sum of 3 skinfolds: triceps, subscapular, suprailiac), Body Mass Index (BMI), Rohrer index, head-to-chest circumference index, Marty's index of chest growth, arm muscles circumference index, cross-section area of arm (mm^2^), cross-section area of arm muscles (mm^2^), cross-section area of arm adipose tissue (mm^2^), head breadth-to-length index, morphological face index, head-to-nose index. Obtained results were analyzed with reference to the Polish norms elaborated by Palczewska & Niedzwiecka [6].

All children with MPS I and II, independently of age and gender, are characterized by severely disordered growth. Both longitudinal and transversal parameters, as body height [B-v), head and neck height [v-sst], trunk length [sst-sy], lower extremity length [B-sy], shoulder breadth [a-a], and hip breadth [ic-ic], correspond to values much below the Polish norms presented in the reference charts (in percentiles) [6-9].

The values of body height in each of the children examined are placed below 3rd centile, which means that they are low-statured (Table 2). Analogically, values related to head and neck height [v-sst] and shoulder breadth [a-a] in all cases are below the 3rd percentile on the Polish reference charts. Hip breadths [ic-ic] in the girl examined (case no. 4) and one boy (case no. 1) are equal to the 3rd percentile, and in both remaining boys (cases no. 2 and 3) this parameter falls below that range. In all three boys the values of lower extremity length [B-sy] and trunk length [sst-sy] are below the 3rd percentile, while in the girl, this parameter is placed in the range between the 3-10th percentile.

**Table 2:** Anthropometric parameters of examined cases

**Case no.**	**1**	**2**	**3**	**4**
Body mass	27,6 kg (3-10c)*	33,2 kg (<3c)	27,5 kg (<3c)	16,7 kg (50–7 5c)
[B-v]	104,7 cm (<3c)	136,7 cm (<3c)	111 cm (<3c)	93,0 cm (<3c)
[B-sy]	57,3 cm (<3c)	70,7 cm (<3c)	58,8 cm (<3c)	45,3 cm (3-10c)
fsst-sy]	30 cm (<3c)	38,8 cm (<3c)	28,8 cm (<3c)	28,7 cm (3-10c)
[a-adIII]	47,2 cm	61.2 cm	41,5 cm	33,0 cm
[a-a]	20 cm (<3c)	25,8 cm (<3c)	24 cm (<3c)	19 cm (<3c)
[ic-ic]	20 cm (3c)	20,3 cm (<3c)	16,5 cm (<3c)	15,5 cm (3c)
[v-sst]	17,4 cm (<3c)	27,2 cm (<3c)	13,4 cm (<3c)	19 cm (<3c)
[g-op]	19 cm	18 cm	19 cm	17,5 cm
[eu-eu]	16 cm	16 cm	15 cm	14,5 cm
[n-gn]	10,5 cm	10,1 cm	10,5 cm	8,5 cm
[zy-zy]	13,5 cm	13,2 cm	14 cm	12 cm
[n-sn]	4 cm	4,2 cm	4 cm	3 cm
[al-al]	4 cm	3,3 cm	3,5 cm	3 cm
HC	55,5 cm (75-90c)	54 cm (25-50c)	58 cm (>97c)	52,5 cm (90-97c)
CC [xi]	64,5 cm (25-50c)	70 cm (25-50c)	65cm (10-25c)	56 cm (90-97c)
AC	22 cm (50-75c)	22,5 cm (25-50c)	20cm (10-25c)	18 cm (90-97c)
Subscapular skinfold	30,3 mm (>97c)	11 mm (75-90c)	16 mm (90-97c)	13 mm (>97c)
Triceps skinfold	20,6 mm (>97c)	12 mm (75-90c)	22 mm (>97c)	19 mm (>97c)
Suprailiac skinfold	30, lmm (>97c)	10 mm (50-75c)	30 mm (>97c)*	15 mm (>97c)
[sst-sv]×l00% [B-v]	28,65 (25-50c) short trunked	28,38 (25- 50c) short trunked	25,94 (<3c) short trunked	30,86 (50-75c) long trunked
[a-a]×l00% [B-v]	19,1 narrow shouldered	18,87 narrow shouldered	21,62 narrow shouldered	20,43 narrow shouldered
[ic-ic]xl00% [B-v]	19,1 platypellia	14,85 dolichopellia	14,86 dolichopellia	16,6 mesopellia
[a-daIII]×l00% [B-v]	45,08 medium upper extremity	44,76 short upper extremity	37,38 short upper extremity	35,48 short upper extremity
[a-r]×l00% [B-v]	21,96 long-armed	30,43 long-armed	17,11 short-armed	20,21 long armed
[a-daIII]×lOO% [B-sy]	80,27 very long-legged	86,56 medium long-legged	70,57 very long-legged	72,84 very long-legged
[B-sy]×l00% [B-vl	54,72 (>97c) short-limbed	51,71 (10-25c) short-limbed	52,97 (50-75c) short-limbed	48,71 (75-90c) short-limbed
[a-a]×l00% [sst-sy]	66,66 (<3c) narrow shoulders	66,49 (<3c) narrow shoulders	83,3 (<3c) broad shoulders	66,20 (<3c) narrow shoulders
[ic-ic]×l00% [sst-sy]	66,66	52,31	57,29	54,00
[ic-ic]×l00% [a-a]	100 (>97c) female proportions	78,68 (>97c) female proportions	68,75 (10-25c) male proportions	81,52 (>97c) medium proportions
BMI	25,17 kg/m2 (90-97c)	17,7 kg/m2 (25-50c)	22,31 kg/m2 (75-90c)	19,3 kg/m2 (>97c)
CC [xi]×l00% [B-v]	61,60 (>97c)	51,20 (90-97c)	58,55 (>97c)	60,21 (>97c)
Global adiposity	81 mm(>97c)	33 mm (75-90c)	68 mm (>97c)	47 mm (>97c)
[v-sst]×l00% [B-v]	16,61 (<3c)	19,89 (>97c)	21,08 (>97c)	20,43 (<3c)
[eu-eu]×l00% [g-op]	84,21 brachycephalus	88,88 hyperbrachy-cephalus	78,94 mesocephalus	82,5 brachycephalus
[n-gn]×l00% [zy-zy]	74,07 hypereuryprosopus	75,75 hypereuryprosopus	75 hypereuryprosopus	70,83 hypereuryprosopus
[al-al]×l00% [n-sn]	100,0 hyperchamaerrhinus	82,5 mesorrhinus	87,5 chamaerrhinus	100,0 hyperchamaerrhinus
MHC×l00% CC[xi]	86,04 (75-90c)	77,14 (75-90c)	89,23 (>97c)	93,75 (10-25c)
Bodv mass (g) [B-v] (cm)	263,6 very weak body build (50-75c)	242,8 very weak body build (3-10c)	247,7 very weak body build (10-25c)	179,6 very weak body build (75-90c)
Bodv mass (g) [B-v] ^3^(cm)^3^	2,4 corpulent (>97c)	1,29 average (3-10c)	2,0 corpulent (>97c)	2,07 corpulent (>97c)
AC cm	15,53 (3-10c)	18,73 (10-25c)	13,09 (<3c)	12,03 (10-25o)
ACS (mm^2^)	3853 (50-75c)	4030 (25-50c)	3185 (10-25c)	2579 (90-97c)
CAM (mm^2^)	1920 (3-10c)	2793 (10-25c)	1364 (<3c)	1152 (3-10c)
CAAT (mm^2^)	1933 (75-90c)	1237 (50-75c)	1821 (75-90c)	1427 (>97c)

**Endnotes:** c – percentile.

The examined children are characterized by some typical body proportions. They are narrow-shouldered (in relation to the values of shoulder-height index and hip-to-shoulder index), short-legged (on the grounds of the values of lower extremity length index), and are of very weak body build (according to classification founded on the Quetelet I index). The values of Marty's index point to a significant growth of the chest: in one boy the value of this parameter falls within the range of 90-97th percentile, and in the other children above the 97th percentile. Analysis of cephalometric values indicates excessive face breadth (hypereuryprosopus) as a characteristic trait of the children. Values of global adiposity are also higher than average (>75c).

Arm muscle circumference (AMC) in the children runs into values much lower than average (<25c). For example, the cross-sectional surface of arm adipose tissue records markedly higher values (>75c).

A short trunk is a distinct characteristic of all the boys with MPS II type, while the girl with MPS I has a relatively long trunk. Interpretation of the remaining indexes of proportion varies with each particular case. Nevertheless, MPS is a factor which should be taken into consideration during differentiation of the cause of a short stature.

## Discussion

In the professional medical literature there is a lack of publications containing detailed anthropometric data relating to the physical growth of children with the MPS spectrum of diseases. Most of the studies concentrate on individual cases and take into consideration only a limited number of anthropometric characteristics. In Poland, for example, several research studies have been performed at the Children's Memorial Health Institute in Warsaw. One of these studies was a long-term examination performed from 1989 to 2009 [7]. The study group consisted of 28 boys with MPS II aged from 0,6 to 27 years. Analysis of results disclosed two phases of physical growth: 1. progressive (during the first 3 years of life, children with MPS II grew faster than the healthy population), 2. pathological (since the end of the third year of life all the measured parameters attained increasingly lower values in comparison to the reference charts). A negative growth trend for all features examined was evidenced by significantly different values from the reference charts.

Similar studies were performed in other European countries, for example, in the Czech Republic and Slovakia [8]. The aim of the research cited was to describe the clinical symptoms, typical course and to evaluate the results of the treatment of 24 Czech and Slovak children with MPS I. Most of the patients had macrocephaly and skeletal changes, cardiomyopathy, hearing impairment, joint stiffness, and carpal tunnel syndrome. The authors emphasized the role of the advanced diagnosis, including the anthropometric parameters. Shah et al. [9] found in a 10-year-old East-Asian boy with MPS such characteristics as an acrocephalic head, short stature, short neck and small stubby fingers with flexion of distal interphalangeal joint, and mild mental retardation. Other authors emphasize that ocular manifestations in MPS I and II may occur not only in the form of corneal clouding, but also include retinopathy, glaucoma, optic disc swelling, optic atrophy, ocular motility and refractive error problems [10].

In practice, the results of anthropometric measurements may be used in the diagnosis of some types of MPS. For example, the phenotype of MPS IVA can be classified into three subgroups according to the patient's final height: severe (<120 cm), intermediate (between 120 and 140 cm) and mild (above 140 cm), respectively [11]. The patients of the severe type are also characterized by features such as short stature, genu odontoid dysplasia, valgum, protrusion of the chest, kyphoscoliosis, hypermobility of joints and abnormal gait, and are mostly below the 90th percentile on the Morquio A growth charts. They often have the onset during infancy, and cannot survive through the second or third decade of life. The patients of the intermediate and mild types, generally called 'attenuated' types, have relatively less skeletal involvement and longer life span [12].

In the cases of our children the values of head breadth-to-length index have revealed three types of proportions: brachycephalic, hyperbrachycephalic and mesocephalic. There was no child with an acrocephalic or dolichocephalic head.

In the group of parameters relating to the process of body growth, special attention must be given to short stature, especially to low values characterizing the length of trunk, head and neck height, lower extremity length, and shoulder and hip breadth. Within the scope of indexes describing the process of differentiation of body proportions, the data obtained point to, in particular narrow shoulders (shoulder growth index), short-legs (relative lower extremity length), and a high level of chest growth (Marty's index).

Children with MPS are characterized by a higher degree of adiposity, which is evidenced by relatively high values of the parameters relating to global adiposity and the cross-sectional area of arm adipose tissue. Children with MPS also display a lower level of musculature development in comparison to their normal peers, as evidenced by the values referring to the cross-sectional area of arm muscles. The boys examined are characterized by a short trunk, whereas the girl is long-statured. This difference is probably determined by gender and the different type of MPS.

On the basis of the morphological face index, it reveals that all examined children are characterized by an excessively broad face. In the group of the parameters describing the growth process, the following typical features were observed: short stature, especially a short trunk, and lower values referring to head and neck height, shoulder and hip breadth. These findings correlate with the results of observations obtained by Shah and colleagues [9-13]. All our children are more than 3-year-old and are characterized not only by a short-stature but also by significantly lower values relating to other aspects of the growth process. Dysmorphic differences are also typical for children with other kinds of diseases such as meningomyelocele [13].

## Conclusions

1. On the basis of anthropometric measurement and various indexes observed it can be concluded that examined children with MPS are characterized by especially low values relating to longitudinal and transversal features of body build.

2. MPS is a disorder which should be taken into consideration during diagnosis of causes of short stature.

3. Some specificity in the formation of body proportionality found in children with MPS could be used in the early diagnosis of this rare disorder.

4. The data presented in Table 2 and final conclusions should not be generalized to all children with MPS but relate to these particular patients.

## Consent

Written informed consent was obtained from the patients’ parents for examination of their children and the use of obtained data for scientific purposes. Also the parents were present during examinations.

## Abbreviations

AC: Arm circumference; ACS: Arm cross-section; AMC: Arm muscle circumference; C: Percentile; CAAT: Cross-section of arm adipose tissue; CAM: Cross-section of arm muscles; CC: Chest circumference; GAGs: Glycosaminoglycals; HC: Head circumference; MHC: Maximal head circumference; MPS: Mucopolysaccharidosis.

## Competing interests

The author declares that has no competing interests.

## Authors’ contributions

LP solely performed all the examinations of the patients, did the literature survey, wrote the first draft, and approved the final manuscript.

## Authors’ information

LP (MD) is a senior lecturer in Medicine in the Institute of Physiotherapy, Faculty of Medicine, University of Rzeszow (Poland).
